# AT THE NEXUS OF PUBLIC POLICY AND AGING: DOCTORAL STUDIES IN GERONTOLOGY AT THE UNIVERSITY OF MASSACHUSETTS BOSTON

**DOI:** 10.1093/geroni/igad104.1773

**Published:** 2023-12-21

**Authors:** Edward Miller, Jeffrey Burr, Kathrin Boerner, Nina Silverstein, Jan Mutchler

**Affiliations:** University of Massachusetts Boston, Boston, Massachusetts, United States; University of Massachusetts Boston, Boston, Massachusetts, United States; University of Massachusetts Boston, Boston, Massachusetts, United States; University of Massachusetts Boston, Boston, Massachusetts, United States; University of Massachusetts Boston, Boston, Massachusetts, United States

## Abstract

One in five PhDs in Gerontology are graduates of the Gerontology doctoral program at the University of Massachusetts (UMass) Boston. The mission of UMass Boston’s program is to provide advanced training and research in gerontology within an interdisciplinary framework drawn primarily, but not exclusively, from the social and behavioral sciences. The program is designed to prepare students for leadership roles as educators, researchers, planners, and policy makers. The program has a deep emphasis on policy development, analysis, and impact, an attribute that goes back to the roots of the Gerontology enterprise at UMass Boston in the late-1970s. This presentation provides an overview of the program’s history and curriculum, along with coursework, research and service-learning experiences, and career paths in policy research and implementation. The program also is committed to increasing diversity in the field of gerontology, with the aim of strengthening policy impact and reducing disparities in the aging experience.

